# Dispositional optimism, pessimism, and psychological distress among Venezuelan migrants in Chile: The role of acculturative stress

**DOI:** 10.1016/j.jmh.2026.100428

**Published:** 2026-07-13

**Authors:** María José Rivera-Baeza, Camila Salazar-Fernández, Belén Salinas-Rehbein, Paola Raipán-Gómez

**Affiliations:** a Departamento de Psicología, Facultad de Ciencias de la Salud, Universidad Católica de Temuco, Manuel Montt 56, Temuco, Chile; b Departamento de Psicología, Facultad de Educación, Ciencias Sociales y Humanidades, Universidad de La Frontera, Temuco, Chile; c Departamento de Psicología, Facultad de Psicología, Universidad de Almería, Almería, España

**Keywords:** Acculturative stress, Optimism, Pessimism, Psychological distress, Migrant mental health

## Abstract

**Introduction:**

Migrants often experience mental health challenges associated with acculturative stress. Dispositional traits, such as optimism and pessimism, may shape how individuals appraise and cope with these challenges, influencing their psychological distress.

**Objective:**

This study aimed to analyse the role of three dimensions of acculturative stress: integration difficulties, nostalgia, and structural barriers, in the relationship between optimism, pessimism and mental health symptoms among Venezuelan migrants living in Chile.

**Method:**

A two-wave longitudinal study was conducted with 223 Venezuelan migrants residing in Chile. Participants completed validated measures of optimism, pessimism, acculturative stress, and psychological distress. A structural equation model was conducted to analyze the data.

**Results:**

Optimism (T1) was negatively associated with integration difficulties (T2) and structural barriers (T2). Pessimism (T1) was positively associated with three dimensions of acculturative stress (T2). Integration difficulties was positively associated with psychological distress (T2). Optimism and Pessimism at Time 1 were not associated with psychological distress (T2). The model explained 43.2% of the variance in psychological distress.

**Discussion:**

These results highlight the importance of both individual dispositions and contextual stressors in understanding the psychological adjustment of migrants. Interventions targeting acculturative stress and fostering adaptive cognitive dispositions may be effective in promoting migrant mental health.

## Highlights

• Pessimism is directly associated with the three dimensions of acculturative stress integration difficulties, nostalgia, and structural barriers.
• Optimism is inversely associated with two dimensions of acculturative stress integration difficulties and structural barriers.
• Only the dimension of integration difficulties is related to psychological distress.

## 1 Introduction

Mental health is a critical global concern, particularly for international migrants who face unique challenges that can affect their psychological well-being. Worldwide, approximately 970 million people live with a mental disorder (World Health Organization, 2021). In the Americas, mental and substance use disorders represent the second leading cause of years lost due to disability and disability-adjusted life years, accounting for 10.5% of the global burden of disease (GBD 2019 Mental Disorders Collaborators, 2022).

According to the World Health Organization, Chile ranks among the countries with the highest burden of mental health disorders globally, with a prevalence of 23% (Vicente et al., 2016). The eleventh edition of the Mental Health Thermometer in Chile, conducted in 2025, reported that 12.7% of respondents exhibited suspected or self-reported symptoms indicative of mental health conditions. Generalized anxiety disorder was identified as the most prevalent condition, affecting 25.8% of the population, and was more frequently reported by women than men. Depression also showed a considerable prevalence of 13%, again with higher rates observed among women (ACHS-UC, 2025).

Despite Chile’s classification as a high-income country—with a GDP per capita of US$15,603 (International Monetary Fund, 2023) and a population of 18.48 million—it faces significant income inequality, with the wealthiest 10% receiving > 50% of the national income (OECD, 2021). This economic disparity contributes to the unequal distribution of mental health disorders, which disproportionately affect certain population groups (Kirkbride et al., 2024). Over the past decade, the country has also experienced a growing influx of migrants from Latin America and the Caribbean, who now represent 8.8% of the national population (Instituto Nacional de Estadísticas, 2024), adding further complexity to the social and health landscape. Some of the reasons that explain the increase in international migration in Chile are related to the political and economic stability, in contrast to other countries in Latin America and the Caribbean (Soto-Alvarado et al., 2022). Most migrants are from within the region—a phenomenon known as South-South migration—and come from Spanish-speaking countries such as Venezuela (41.6%), Peru (14.5%), Colombia (12.3%), and Bolivia (10.4%). An exception is migrants from Haiti (5%), who primarily speak Haitian Creole and French (Instituto Nacional de Estadísticas, 2024).

International migrants are particularly vulnerable to mental health issues due to the numerous challenges associated with the migratory process—such as cultural adjustment to a new context and interaction with the systems of the receiving society—which are associated with a higher incidence of mental health symptoms, including stress, anxiety, and depression (Cabieses et al., 2024; Horne, 2024). Several studies conducted in Chile with international migrants have shown symptoms of mental health disorders. For example, a study conducted with international migrants from Venezuela, Colombia, and Haiti living in southern Chile found high levels of psychological stress and anxiety symptoms on the DASS-21 scale (Barrera-Herrera et al., 2023). Similar results were found in the study of Baeza-Rivera et al. (2022), where 72% of participants experienced symptoms of stress, 62% symptoms of anxiety, and 46% symptoms of depression.

Considering the extent of mental health symptomatology among migrants and the difficulties and challenges of the migration process in the receiving society, it is important to explore individual characteristics that may act as protective or risk factors for their mental health. Optimism-pessimism, the tendency to maintain a positive or negative outlook during adversity and to expect favorable or unfavorable outcomes in the future (Ho et al., 2010), are dispositional traits that have been studied for its association with psychological well-being and health outcomes (Hanssen et al., 2015). According to the expectancy-value theory of motivation, an individual's motivation is determined by their perceived probability of success and the subjective value they assign to the task or its anticipated outcome (Scheier and Carver, 1985). Consistent with the premises of the model, it has been argued that optimists’ positive expectations about the future foster sustained effort toward goal attainment, even in the face of adversity. Conversely, pessimists, due to their uncertainty regarding future outcomes, are more likely to disengage from goal pursuit when confronted with adversity (Carver et al., 2010).

Both optimism and pessimism encompass nuances that may be particularly relevant in the context of health. In this regard, the concept of *illusory optimism* (Weinstein, 1982) posits that individuals tend to overestimate the likelihood of positive events while underestimating the probability of negative ones. This perspective challenges the notion that optimism is inherently adaptive, as expectations grounded in unrealistic assessments of circumstances may lead to unnecessary risk exposure or inadequate preparation for adverse situations (Gassen et al., 2021). Conversely, pessimism may not be as detrimental as commonly perceived. A moderate degree of pessimism can be functional, as it entails evaluating a range of potential scenarios, thereby fostering preparedness for adversity. This anticipatory process enables individuals to foresee potential difficulties and develop more effective coping strategies. Accordingly, those with less favourable expectations may be better equipped to manage negative situations, having already anticipated such outcomes (Atta et al., 2024; Adams, 2023).

In line with these theoretical perspectives, research indicates that optimism is associated with greater emotional well-being, reduced vulnerability to depressive symptoms, and a greater tendency to employ adaptive coping skills in the face of stress (Armbruster et al., 2015; Ho et al., 2010). In contrast, evidence shows that pessimistic individuals are at higher risk for depressive symptoms (Karhu et al., 2024). Similar results have been found in migrant populations. For example, a study conducted with 421 Latin American migrants living in the United States found that higher levels of optimism among men were associated with fewer symptoms of anxiety, depression, and stress (Camacho de Anda and Becerra, 2023). In Chile, the available evidence regarding optimisms among migrants has been limited to Colombian migrant populations. For example, a study conducted with 919 adult Colombian migrants living in Chile, showed that higher optimism was associated with fewer anxiety symptoms (Urzúa et al., 2023). Regarding pessimism, a study conducted with 572 Latino college students living in the United States found that those with greater levels of pessimism exhibited more depressive symptoms and suicidal behaviors (Chang, 2024). In Chile, as far as the authors have reviewed, there are no studies available that have addressed the association between pessimism and mental health symptoms in Latin American migrants.

Empirical evidence indicates a direct association between optimism–pessimism and mental health outcomes among migrant populations. The available literature on migrant population in Chile remains limited in certain areas, particularly regarding studies that allow for the examination of migratory processes and their effects on health and well-being. In this regard, the predominance of cross-sectional designs restricts the understanding of individuals’ experiences throughout the migratory process over time. Additionally, the specific variables that may help comprehend this relationship within the broader context of migratory processes remain insufficiently understood. In line with this, several studies have shown that acculturative stress, defined as the stress resulting from the demands and difficulties of cultural adaptation, which often exceed individuals’ personal and social resources, is a multidimensional process that may involve different sources of stress, including nostalgia for the country of origin, structural barriers in the host society, and difficulties in social and cultural integration. These dimensions may be particularly relevant for understanding how migratory experiences are associated with psychological distress. This makes it a relevant psychological factor for understanding emotional discomfort among migrant populations. For instance, Urzúa et al. (2021) in a cross-sectional study argued that acculturative stress mediated the relationship between experiences of ethnic and racial discrimination and both physical and mental health among Colombian migrants residing in Chile. This body of evidence supports the claim that acculturative stress not only is associated with psychological distress but also could explain how individual or contextual factors affect mental health in migrant populations.

Furthermore, the present study proposes that acculturative stress operates as a theoretically plausible pathway between cognitive dispositions—optimism and pessimism—and symptoms of psychological distress. While prior literature supports direct relationships between these variables, little is known about the potential processes through which dispositional traits may be linked to psychological distress.

Research indicates that optimism-pessimism is associated with acculturative stress in migrants. A cross-sectional study conducted with 1027 migrant college students in China demonstrated that higher levels of optimism were associated with lower levels of acculturative stress (Chen et al., 2019). Similar findings were reported in a study by Yakunina et al. (2013), which found that greater optimism was associated to reduced acculturative stress and better adjustment to the host society. Alkış (2014), for example, explored the relationship between optimism and acculturative stress among international students, finding that higher levels of optimism were associated with lower levels of acculturative stress. These findings suggest that optimism may serve a protective role in the face of cultural challenges. Taken together, this evidence suggest that acculturative stress may represent a theoretically relevant explanatory pathway linking dispositional traits, such as optimism and pessimism, to psychological distress in migrant population. Some of the situations that have been studied as stressors in the acculturation process of migrants include language barriers, experiences of discrimination, unemployment, and difficulties in accessing healthcare and other essential services (Avaria et al., 2022; Baeza-Rivera et al., 2024; Cabieses et al., 2021, 2023; Mera-Lemp et al., 2019). For this study, three dimensions will be considered to conceptualized acculturative stress: feelings of nostalgia for one’s country and social ties left behind (intrapersonal), challenges in establishing meaningful connections within the host society (interpersonal), and systemic barriers embedded within the institutions and policies of the host country (socio-structural) (Baeza-Rivera et al., 2022).

Acculturative stress, in turn, has been associated with mental health issues in multiple studies. For example, a cross-sectional study of 257 Mexican immigrant women found that acculturative stress was directly associated with greater psychological distress (Bekteshi, 2024). Comparable results were reported in a systematic review by Lerias et al. (2024), which concluded that Latino immigrants living in the United States experienced higher psychological distress as a result of acculturative stress. Longitudinal evidence also supports this association; a study by Allen et al. (2024), conducted with 562 children of immigrants, found that acculturative stress predicted depression over a 15-year period. Although acculturative stress has been consistently linked to negative mental health outcomes, individual-level dispositions may shape how migrants experience and cope with such stress.

## 2 The present study

This study aims to analyze the role of three dimensions of acculturative stress- nostalgic, structural barriers, and integration difficulties- in the relationship between optimism-pessimism and mental health symptoms among Venezuelan migrants living in Chile. We hypothesize that dispositional traits such as optimism and pessimism may shape how migrants appraise and respond to the challenges of cultural adaptation, potentially increasing or decreasing their vulnerability to acculturative stress and, in turn, psychological distress. To test this, we examined whether the relationship between dispositional traits (optimism and pessimism in T1) and psychological distress (T2) was indirectly explained through three dimensions of acculturative stress (T2): nostalgia, structural barriers, and adaptation difficulties. While prior research has documented cross-sectional associations between acculturative stress and mental health outcomes, few studies have explored these processes over time, and none, to our knowledge, have done so in the south-south migration context of Venezuelan migrants in Chile.

We will examine these associations among Venezuelan migrants living in Chile. This group constitutes a particularly relevant case because Venezuelan immigrants currently face some of the highest levels of social distance and prejudice among Chileans, with recent survey evidence placing them as the most negatively evaluated migrant group by nationality of origin (Activa Research, 2025). This social reception context may contribute to a distinctive acculturative experience compared with other migrant groups in Chile.

According to data from the National Migration Survey and the National Institute of Statistics (INE, 2024), most Venezuelan migrants in Chile are of economically active age, predominantly between 18 and 40 years old. Venezuelan migration has been largely driven by the economic crisis in their country of origin, while family reunification has been identified as one of the main reasons for choosing Chile as a destination country. In terms of educational profile, Venezuelan migrants tend to present relatively high educational attainment levels: 64.6% have completed tertiary education, 30.8% have completed secondary education, and only a small proportion (4.6%) report primary education or fewer years of schooling. Among those with university education, many belong to engineering and technology fields. However, despite these qualifications, an important challenge concerns the recognition and homologation of academic degrees and professional credentials in Chile, a process that only a minority of migrants have successfully completed.

This study was conducted in the La Araucanía Region because, although the first waves of Venezuelan migration in Chile were primarily concentrated in north and central regions of the country, subsequent internal mobility processes, referred to as domestic migration, involving changes of residence between regions within the same country, have progressively increased the presence of Venezuelans in southern regions of Chile such as La Araucanía (Instituto Nacional de Estadísticas, 2024). This region constitutes a particularly relevant context due to its sociodemographic conditions; the income poverty rate stands at 28.6%, which is substantially higher than the national average of 17.3% (Ministerio de Desarrollo Social y Familia, 2026). Regarding intercultural dynamics, 34.5% of its inhabitants identify as belonging to an Indigenous people. Additionally, although migrants represent approximately 2.1% of the regional population, the majority of this migrant population is of Venezuelan origin. These characteristics, together with the region’s intercultural dynamics and emerging challenges related to migrant integration and access to opportunities, provide a unique context for examining the experiences of Venezuelan migrants in Chile. Considering these contextual characteristics and the predominance of Venezuelan migrants in La Araucanía, the analyses focused exclusively on this group, allowing for a more specific and culturally contextualized understanding of the relationships examined in this study.

## 3 Method

### 3.1 Participants

This study is part of a larger research project that included migrants from Venezuela, Colombia, and Haiti. However, for the purposes of the present study, only Venezuelan migrants were considered, as they represented > 85% of the total sample. Selected through purposive snowball sampling a total of 223 migrant participants with complete data in both the first and second wave of the study were included (Time 1, March–September 2021, Time 2, February–June 2022). The time between T1 data collection and the beginning of T2 was approximately eleven months. The inclusion criteria were: (a) being over 18 years old; (b) residing in the *La Araucanía* region. Individuals who were in the country for tourism purposes were excluded from the study. The composition and description of the sample are presented in Table 1.

**Table 1:** Description of the participants.

Variable	M (SD) or n (%)
Age	34.4 (9.5)
Time living in Chile (months)	47.0 (80.2)
Sex	
Male	75 (33.6)
Female	148 (66.4)
Marital status	
Single	107 (48.0)
Married	48 (21.5)
Divorced	1 (0.4)
Widowed	5 (2.2)
De facto separated	5 (2.2)
Cohabiting	57 (25.6)
Education level	
Primary school	6 (2.7)
Secondary school	50 (22.4)
University	149 (66.8)
Postgraduate	18 (8.1)
Socioeconomic status	
Very low-low	126 (56.5)
Middle	92 (41.3)
High-very high	5 (2.2)

*Note*. N = 223. Continuous variables are presented as M (SD), and categorical variables are presented as n (%). For time living in Chile, valid n = 220 due to three missing values. Percentages are based on valid cases and rounded to one decimal place. Educational level and socioeconomic status were grouped into broader categories.

### 3.2 Instruments

Participants completed a self-report questionnaire that included sociodemographic variables and the following scales:

**Dispositional Optimism.** Optimism and pessimism were assessed using the Spanish version of the Life Orientation Test-Revised (LOT-R) (Valdelamar-Jiménez, and Sánchez-Pedraza, 2017). The scale includes 10 items rated Likert scale from 1 (Strongly disagree) to 5 (Strongly agree), with two subscales: Optimism (e.g., “In uncertain time, I usually expect the best”) and Pessimism (e.g., “I rarely expect things to go my way”), each comprising three items. Four additional items serve as distractors and are not used in the analysis. The reliability estimates for optimism (α = 0.82) and pessimism (α = 0.70) were adequate.
**Acculturative Stress.** The Acculturative Stress Scale (EEA) by Baeza-Rivera et al. (2024) was used, which contains 25 items rated on a frequency scale from 0 (Never) to 3 (Always). This scale is designed to assess intrapersonal, interpersonal, and socio-structural aspects, where higher scores indicate a greater presence of acculturative stress. The items were grouped into three factors: Factor 1: Integration difficulties (α = 0.93), Factor 2: Nostalgia for the country of origin (α = 0.87), Factor 3: Structural barriers to migration (α = 0.91). For the CFA and SEM analyses, the integration difficulties and structural barriers factors were modeled using three theoretically defined item parcels each. For integration difficulties, the parcels represented: (1) adaptation and belonging, (2) cultural tension with the host society, and (3) negative future expectations. For structural barriers, three parcels represented: (1) rights and institutional access, (2) material, labor and housing integration, and (3) regularization and migratory insecurity. The nostalgia factor was modeled using its original three items as indicators, given that this dimension already contained three items. These items were not framed within a restricted recent time window; rather, they assess broader acculturative experiences related to the experience of migrating to Chile.
**Psychological Distress.** was measured with the validated Spanish version of the Depression, Anxiety, and Stress Scale (DASS-21) (Antúnez and Vinet, 2012). This 21 -item scale assesses the extent to which participants experienced symptoms during the last week, making psychological distress the most temporally proximal construct in the model. Responses were rated on a 4-point Likert frequency scale (0 = did not apply to me at all, 3 = applied to me very much or most of the time) and consists of three subscales: Depression (e.g., “I couldn't feel anything positive”), Anxiety (e.g., “I noticed that my mouth was dry”), and Stress (e.g., “I found it very difficult to calm myself down”). Internal consistency was excellent: Depression (ω = .89), Anxiety (ω = .88), and Stress (ω = .90). In the CFA and SEM analyses, psychological distress was modeled as a latent factor using the mean scores of the Depression, Anxiety, and Stress subscales as three indicators.
**Sociodemographic Questionnaire**. To characterize the sample, participants self-reported their age in years, time living in Chile in months, sex, marital status, education level, and socioeconomic status (SES). SES was measured using the MacArthur Scale of Subjective Social Status (Adler et al., 2000). While the original scale presents a ten-rung ladder, the present study employed a five-rung version adapted to reflect Chile`s socioeconomic classification system. This instrument has been used in previous research (Bryan et al., 2024), which coincides with the socioeconomic classification system that exists in Chile. The Asociación de Investigadores de Mercado (AIM) has historically organized the population into five operative socioeconomic segments-ABC1 (High), C2 (upper middle), C3 (middle), D (lower-middle/vulnerable), and E (low/poverty)- representing the most widely used stratification framework in the country (Asociación de Investigadores de Mercado (AIM Chile), 2018). The adapted ladder thus included five-rung corresponding to the levels very low, low, middle, high and very high, allowing participants to locate themselves within a classification structure that is both culturally meaningful and broadly recognized in Chilean society.

Participants were shown the ladder and asked to place themselves on the step that best reflected their perceived social standing relative to others. The instructions indicated to the participants that at the top of the ladder represented individuals with greater access to economic and educational resources and higher-status occupations, whereas the bottom represented individuals with fewer resources and lower-status occupations. This measure was intended to capture participants’ subjective perception of their socioeconomic position rather than relying exclusively on objective indicators such as income or occupation.

### 3.3 Procedure

Participants were recruited through migrant organizations in the Araucanía Region, which disseminated the study invitation via social media, mailing lists, and direct contact with community leaders. Data were collected through a secure online survey platform. Before participating, individuals accessed a digital informed consent form—approved by the Ethics Committee of the authors’ university—which explained the study’s objectives, procedures, and ethical safeguards, including confidentiality, voluntary participation, protection of participants’ integrity, and mechanisms to ensure their psychological well-being. Only those who accepted the terms of the informed consent were granted access to the survey. Completing the questionnaire took approximately 20 min. Participants received financial compensation of CLP 7000 (≈ 8 USD) for completing Time 1 and CLP 10,000 (≈ 12 USD) for Time 2.

### 3.4 Data analysis

We first conducted exploratory analyses for each variable of interest to detect missing data and examine their distributions, ensuring they were suitable for inclusion in multivariate procedures. Descriptive statistics and bivariate correlations were also inspected prior to estimating the measurement and structural models.

### 3.5 Measurement model

We conducted a confirmatory factor analysis (CFA) to evaluate the measurement structure of the scales used in the study. Specifically, we estimated a six–factor measurement model including optimism, pessimism, integration difficulties, nostalgia, structural barriers, and psychological distress. CFA was used to examine whether the hypothesized measurement structure was consistent with the empirical covariance structure of the data, following standard recommendations for the evaluation of latent–variable measurement models (Kline, 2016).

Because most constructs were measured with only three indicators, the corresponding single–factor CFA models were just–identified and therefore do not provide informative goodness–of–fit indices. Accordingly, we report the fit of the full six–factor measurement model, together with evidence of convergent and discriminant validity. Convergent validity was assessed using standardized factor loadings, composite reliability (CR), and average variance extracted (AVE). Factor loadings of .40 or higher were considered minimally acceptable, CR values above .60 were considered acceptable for brief scales, and AVE values of .50 or higher were considered evidence of adequate convergent validity (Bagozzi and Yi, 1988). Discriminant validity was evaluated using the heterotrait-monotrait ratio of correlations (HTMT), with values below .85 indicating adequate discriminant validity (Henseler et al., 2015).

### 3.6 Model estimation

The CFA and structural equation models were estimated using robust maximum likelihood estimation with robust standard errors (MLR). Although WLSMV is often recommended for CFA and SEM models estimated directly from item–level ordinal indicators with few response categories, the present model included several composite indicators computed as mean scores from multiple items. These composite indicators had a larger number of possible values than the original Likert–type items and were therefore treated as approximately continuous.

Because Mardia’s test indicated violations of multivariate normality (*p* < .05), MLR was selected because it provides robust standard errors and scaled test statistics under non–normality. As a robustness check, we attempted to estimate the model using WLSMV; however, this model did not yield an admissible solution, producing a non–positive definite covariance matrix. Therefore, the MLR solution was retained for the main analyses.

### 3.7 Structural model specification

The hypothesized structural equation model examined the associations between optimism (T1) and pessimism (T1) and psychological distress (T2) through acculturative nostalgia, acculturative difficulties, and acculturative barriers (T2). Indirect effects from optimism and pessimism to psychological distress through acculturative stress were estimated to test the proposed mediation pathways.

To account for variability related to the heterogeneous backgrounds of migrant participants, the model controlled for psychological distress in the previous time, gender, and months living in Chile.

### 3.8 Model fit

All models were estimated using the *lavaan* package (Rosseel, 2012) in R software. Model fit was assessed using multiple indices: Chi-square (χ²), Comparative Fit Index (CFI), Tucker-Lewis Index (TLI), Standardized Root Mean Square Residual (SRMR), and Root Mean Square Error of Approximation (RMSEA) with a 90% confidence interval. Established cut-offs were applied to evaluate adequacy of fit, with CFI and TLI values above .90 and SRMR and RMSEA values of .08 or lower considered acceptable (Hu and Bentler, 1999).

### 3.9 Sensitivity analysis

To address concerns regarding the temporal ordering of the mediation model, we estimated an alternative model with a more temporally conservative specification. In this sensitivity analysis, optimism, pessimism, acculturative nostalgia, integration difficulties, and structural barriers were specified as Time 1 variables, whereas psychological distress was specified as the Time 2 outcome. This alternative model allowed us to examine whether the proposed indirect associations were supported when all predictors and mediators were modeled as preceding psychological distress.

We did not estimate reverse–order models in which psychological distress predicted acculturative stress dimensions, because psychological distress was assessed with reference to recent symptoms during the last week, whereas acculturative stress was measured as broader migration–related experiences without a restricted recent time window. Therefore, psychological distress was considered the most temporally proximal construct in the model. This alternative specification was interpreted as a sensitivity analysis rather than as a primary substantive model.

## 4 Results

Descriptive statistics and bivariate correlations for all variables in the model are reported in Table 2.

**Table 2:** Correlation between the study variables.

	1	2	3	4	5	6	7	8
1. Optimism (T1)	–							
2. Pessimism (T1)	.20^⁎⁎^	–						
3. Psychological distress — depression (T2)	–.11	.23^⁎⁎⁎^	–					
4. Psychological distress — stress (T2)	–.11	.23^⁎⁎⁎^	84^⁎⁎⁎^	–				
5. Psychological distress — anxiety (T2)	–.15*	.24^⁎⁎⁎^	.82^⁎⁎⁎^	.83^⁎⁎⁎^	–			
6. Acculturative stress — factor 1: integration difficulties (T2)	–.05	.20^⁎⁎^	.37^⁎⁎⁎^	.41^⁎⁎⁎^	.35^⁎⁎⁎^	–		
7. Acculturative stress — factor 2: nostalgia (T2)	–.03	.17*	.23^⁎⁎⁎^	.25^⁎⁎⁎^	.22^⁎⁎^	.47^⁎⁎⁎^	–	
8. Acculturative stress — factor 3: structural barriers (T2)	–.07	.17*	.44^⁎⁎⁎^	.40^⁎⁎⁎^	.35^⁎⁎⁎^	.48^⁎⁎⁎^	.21^⁎⁎^	–

*Note*. Pearson’s correlations are reported. *p < .05. ^⁎⁎^*p* < .01. ^⁎⁎⁎^*p* < .001.

### 4.1 Measurement models, convergent and discriminant validity

The six–factor CFA model showed good fit to the data: χ2(120) = 206.313, *p <* .001, CFI = .952, TLI = .941, RMSEA = .057 [.043, .070], SRMR = .063. Standardized factor loadings were all statistically significant and above .40, ranging from .429 to .930 (see Table 3), providing initial support for the measurement construct. CR values ranged from .632 to .936, indicating acceptable to excellent internal consistency, particularly considering the brief length of the scales. AVE values ranged from .389 to .830. AVE values were above the recommended .50 threshold for most constructs, with the exception of pessimism and acculturative nostalgia, which showed comparatively lower convergent validity. Nevertheless, these constructs were retained because their factor loadings were theoretically coherent, their CR values were acceptable, and they correspond to previously validated measures. Discriminant validity was supported by HTMT ratios below .85 for all construct pairs, consistent with the criterion proposed by Henseler et al. (2015). Overall, these results provide adequate support for the use of the measures in the subsequent structural equation models. Detailed results are presented in Table 3.

**Table 3:** Factor loadings, composite reliability, average variance extracted, and HTMT ratios for the study measures.

Constructs	Factor loading	Composite reliability (CR)	Average variance extracted (AVE)	Hetero-Trait-Mono-Trait index (HTMT)
Optimism (T1)				Optimism – Pessimism = .216
In uncertain times, I usually expect the best.	.789*	.822	.607	
I am always optimistic about my future	.805*			
Overall, I expect more good things to happen to me than bad	.742*			
Pessimism (T1)				
If something bad can happen, it will probably happen to me.	.476*	.632	.389	
I rarely expect things to go my way.	.429*			
Overall, I expect more good things to happen to me than bad.	.742*			
Acculturative stress: Nostalgia (T2)				
I feel bad when I think about everything, I left behind in my home country.	.795*	.715	.462	Nostalgia – Integration difficulties = .854
I miss the customs of my country.	.538*			
I feel bad about how my decision to emigrate affected my loved ones.	.682*			
Acculturative stress: Structural barriers (T2)				Nostalgia – Structural barriers = .414
Parcel 1: Rights and institutional access	.698*	.788	.557	
Parcel 2: Material, labor, and housing integration	.863*			
Parcel 3: Regularization and migratory insecurity	.663*			
Acculturative stress: Integration difficulties (T2)				Integration difficulties – Structural barriers = .739
Parcel 1: Adaptation and belonging	.872*	.867	.686	
Parcel 2: Cultural tension with the host society	.812*			
Parcel 3: Negative future expectations	.799*			
Psychological distress (T2)				
Anxiety	.891*	.936	.830	Unifactorial construct
Stress	.930*			
Depression	.911*			

*Note*. * *p* <.05.

### 4.2 Structural models

**Fig. 1:**
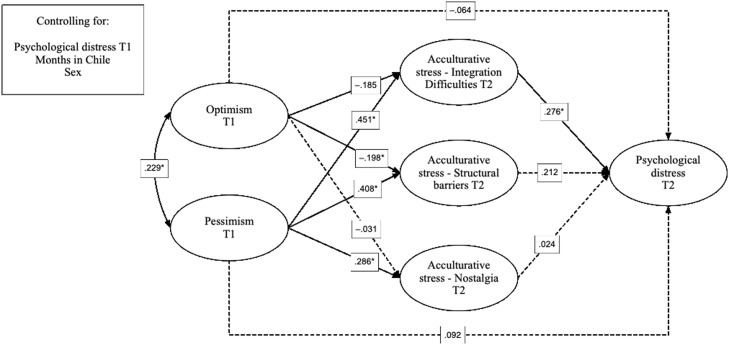
*Note*. * *p* < .05, ** *p* < .01, *** *p* < .001*.* Dashed lines in Fig. 1 indicate a non-significant association, and continuous lines indicate a significant association.

The proposed SEM showed acceptable fit to the data, χ2(211) = 408.527, *p <* .001, CFI = .915, TLI = .900, RMSEA = .067 [.057, .077], SRMR = .079. Fig. 1 presents the standardized coefficients of the structural model.

As shown in Fig. 1, optimism (T1) was negatively associated with integration difficulties (T2) (β = –.185, *p =* .018), and structural barriers (T2) (β = –.198, *p =* .013). However, optimism (T1) was not significantly associated with nostalgia (T2) (β = –.031, *p =* .750). In contrast, pessimism (T1) was positively associated with the three dimensions of acculturative stress (T2): integration difficulties (β = .451, *p <* .001); structural barriers (β = .408, *p =* .001); and nostalgia (β = .286, *p =* .017).

Regarding the associations between acculturative stress (T2) and psychological distress (T2), integration difficulties were positively associated with psychological distress (β = .276, *p =* .040). The association between structural barriers and psychological distress was positively but did not reach conventional levels of statistical significance (β = .212, *p =* .079). Nostalgia was not significantly associated with psychological distress (T2) (β = .024, *p =* .769). Baseline psychological distress was positively associated with psychological distress (T2) (β = .285, *p =* .002). Months in Chile and reported sex of the participants did not showed significantly associations with psychological distress (*ps >* .844). The direct effects of optimism, β = –.064, *p =* .415, and pessimism, β = .092, *p =* .454, on psychological distress (T2) were not statistically significant after accounting for the acculturative stress dimensions, baseline psychological distress, and covariates.

The model explained 20.0% of the variance in integration difficulties, 7.9% of the variance in nostalgia, 16.9% of the variance in structural barriers, and 43.2% of the variance in Time 2 psychological distress.

The specific indirect effects from optimism to psychological distress through each acculturative stress dimensions were not statistically significant when considered separately: integration difficulties, β = –.051, *p =* .130; structural barriers, β = –.042, *p =* .134; and nostalgia, β = –.001, *p =* .825. However, the total indirect effect of optimism through the three acculturative dimensions was statistically significant, β = –.094, *p =* .014. Similarly, the specific indirect effects from pessimism to psychological distress through each acculturative stress dimension were not statistically significant when considered separately: integration difficulties, β = .125, *p =* .079; structural barriers, β = .086, *p =* .130; and nostalgia, β = .007, *p =* .769. Nevertheless, the total indirect effect of pessimism through the three dimensions of acculturative stress was statistically significant, β =.218, *p =* .002. These results suggest that optimism and pessimism were indirectly associated with psychological distress through their overall associations with acculturative stress, although no single acculturative stress dimension independently accounted for the indirect effect.

### 4.3 Sensitivity analysis

To address concerns regarding the temporal ordering of the mediation model, we estimated an alternative model with a more temporally conservative specification. In this sensitivity analysis, optimism, pessimism, integration difficulties, structural barriers and nostalgia were specified as Time 1 variables, whereas psychological distress was specified at T2. Although this model converged and showed acceptable fit, χ2(171) = 377.715, *p <* .001, CFI = .915, TLI = .895, RMSEA = .074 [.064, .084], SRMR = .070, it produced unstable estimates, including standardized regression coefficients greater than 1.0 for the direct effects of optimism and pessimism (T1) on psychological distress (T2), as well as large standard errors. Substantively, this alternative specification did not provide evidence for a prospective mediation process. None of the specific indirect effects were statistically significant (total indirect effects of optimism, *b* = .064, 95% CI [–.196, .324], *p* =.627, and pessimism, *b* = –.148, 95% CI [–1.839, .956], *p* =.536). In addition, none of the acculturative stress dimensions (T1) were significantly associated with psychological distress (T2), all *ps >* .160. Therefore, this alternative specification was interpreted as sensitivity analysis and did not support a robust mediation process.

## 5 Discussion

This study aimed to analyze the role of three dimensions of acculturative stress, integration difficulties, nostalgia, and structural barriers in the relationship between optimism, pessimism and mental health symptoms among Venezuelan migrants living in Chile. The findings were generally consistent with the hypothesized model, suggesting that integration difficulties were associated with the relationship between dispositional characteristics and psychological distress, accounting for 43.2% of the variance in psychological distress. These results highlight the importance of both individual dispositions and contextual stressors in understanding the psychological adjustment of migrants.

The proposed model examined the associations between dispositional optimism and pessimism at Time 1, the three dimensions of acculturative stress at Time 2 (integration difficulties, nostalgia, and structural barriers), and psychological distress at Time 2. The findings showed that pessimism was positively and significantly associated with higher levels of the three dimensions of the acculturative stress, and only the integration difficulties significantly associated with greater psychological distress. In contrast, optimism only show significant negative associations with structural barriers and integration difficulties. Direct paths from optimism and pessimism to psychological distress were also examined; however, these associations were not statiscally significant. Overall, the findings suggests that the integration difficulties were associated with the relationship between pessimism, and optimism and psychological distress.

From a theoretical perspective, these results can be understood through the transactional model of stress and coping proposed by Lazarus and Folkman (1984), which posits that stress results from the interaction between an individual and their environment are mediated by cognitive appraisal. In this context, pessimism may lead individuals to perceive environmental demands as more threatening and their own resources as insufficient, thereby intensifying the experience of acculturative stress, considering the uncontrollability and unpredictability, as well as the difficulties of integration and structural barriers. Supporting this interpretation, Chang (2002) found that pessimism exacerbated the relationship between perceived stress and psychological maladjustment, suggesting that a negative cognitive orientation (e.g., pessimism) increases emotional vulnerability in demanding contexts such as migration. Thus, these findings do not appear to support the notion of defensive pessimism, which would imply that pessimism is being used as an adaptive cognitive strategy (i.e., for planning and preparation). Rather, it seems to reflect a generalized negative orientation that increases emotional vulnerability and threat perception (Atta et al., 2024; Adams, 2023).

Interestingly, optimism was significantly associated with lower levels of integration difficulties and structural barriers, suggesting that positive future expectations may play a protective role in specific domains of the acculturation process. In particular, optimism may facilitate coping with challenges related to adaptation, sense of belonging, and perceived barriers linked to institutional access, regularization processes, and social integration. However, optimism was not associated with nostalgia, indicating that its protective effects may be context-dependent and limited when migrants face broader structural or emotional challenges associated with migration experiences. The findings are consistent with previous literature suggesting that the benefits of optimism are often strengthened when accompanied by supportive social and contextual resources (Carver et al., 2010; Scheier & Carver, 1992). Based on these findings, it is important that strategies aimed at promoting migrant well-being consider both individuals internal coping resources and the external resources available withing the host society. Internal resources may include dispositional tendencies such as optimism and pessimism, as well as individual coping styles and emotional regulation capacities. At the same time, external resources as social support, opportunities for social integration, and access to institutional services may play a central role in facilitating adaptation and psychological well-being. In this regard, low-intensity psychological interventions such as Problem Management Plus (PM+), developed by the World Health Organization, may represent a promising approach for migrant populations exposed to adversity. These interventions aim not only to reduce symptoms of psychological distress, but also to strengthen coping capacities, problem-solving skills, emotional regulation, and the effective use of social support networks (Schäfer et al., 2023). Supporting this perspective, a mixed-method study conducted with Venezuelan migrants and refugees in Colombia found that a culturally adapted version of PM+ was associated with significant improvements in subjective well-being, quality of life, and self-identified problems. The study further highlighted the importance of integrating mental health interventions with social and humanitarian support systems, particularly in populations exposed to multiple stressors related to migration, economic insecurity, discriminations, and limited access to services (Perera et al., 2022). Because internal and external resources interact dynamically, interventions should move beyond approaches focused solely on individual positive expectations, and instead adopt a broader perspective that simultaneously promotes personal coping resources and supportive social and institutional environments within the host society.

The current findings also reinforce the relevance of acculturative stress dimensions in understanding psychological distress among Venezuelan migrants. This is consistent with previous research, including Bekteshi (2024), who reported similar results among Mexican immigrant women in the U.S., and Baeza-Rivera et al. (2022), who found that acculturative stress predicted mental health symptoms among migrants in Chile. Similarly, Urzúa et al. (2016, 2021) highlighted the mediating role of acculturative stress in the relationship between ethnic discrimination and mental health in South American migrants.

In this study, multiple dimensions of acculturative stress were assessed, including integration difficulties, nostalgia, and structural barriers. These are among the most commonly reported stressors in the migration literature and were shown to contribute to increased psychological distress in the South-South migration context (Baeza-Rivera et al., 2024). These findings underline the need for integrated psychological and social interventions that address intrapersonal, interpersonal, and socio-structural factors affecting migrant mental health. Evidence suggests that interventions promoting cultural integration—preserving heritage culture while adopting aspects of the host culture—can have a positive impact on mental health (Choy et al., 2021). Factors such as strong ethnic identity, and community belonging (Lashari et al., 2023), as well as improved access to services (Lardier et al., 2023), have been linked to reduced psychological distress and enhanced personal development in migrant populations (Benet-Martínez, 2012; Zimmermann et al., 2021).

A key component of acculturative stress is discrimination, both interpersonal and institutional, which contribute to increased anxiety and depression and decreased well-being (Lahoz i Ubach and Forns i Santacana, 2016; Schmitt et al., 2014; Yoo and Lee, 2009). Although discrimination was not directly assessed in the present study, its established role in the development of acculturative stress highlights the importance of addressing it in future research. Interventions should aim to foster tolerance and reduce prejudice to mitigate perceived discrimination. Similarly, social support has been widely recognized as a protective factor against acculturative stress (Berry, 1997; Zimmermann et al., 2021). While not measured here, future studies should consider its role in moderating the psychological impacts of migration. Programs that strengthen social networks may help buffer these effects and enhance migrants’ overall well-being (Baeza-Rivera et al., 2022; Li et al., 2016; Song et al., 2024).

To address mental health challenges among migrant populations, it is essential to consider multi-level strategies that go beyond individual-focused interventions. Socio-community approaches, such as programs that foster social integration, reduce discrimination, and strengthen community networks, can create supportive environments that mitigate risk factors and promote psychological well-being (European Commission, 2024). Community-based initiatives offering psychoeducation and group activities—such as meditation, physical exercise, or cultural exchange—can enhance group cohesion and a sense of belonging, which are key protective factors for migrant mental health (Kim et al., 2021). While individual-level interventions such as cognitive-behavioral therapy have shown effectiveness in reducing mental health symptoms, culturally adapted psychotherapeutic approaches are needed to approach all the diverse challenges migrants are face with in a new host society (Kananian et al., 2020). However, their implementation should be considered complementary to broader structural and community-based efforts to ensure sustainable and equitable mental health support for migrant populations.

This study also highlights the importance of structural conditions in shaping migrants' mental health. International organizations such as the WHO have emphasized that legal insecurity, discrimination, and limited access to basic services elevate psychological vulnerability among migrants. Public policies should promote social inclusion and ensure access to culturally sensitive mental health services through community-based strategies that directly address structural stressors (World Health Organization, 2025). In this sense, interventions aimed at promoting migrant well-being should not focus exclusively on dispositional traits, such as optimism, but should also seek to reduce structural barriers, strengthen social support, and improve access to institutional resources in the host society.

Several limitations of this study should be acknowledged. First, the exclusive use of self-report measures may have introduced social desirability and perception biases. Second, the study relied on a non-probabilistic snowball sampling strategy, which limits the generalizability of the findings. This recruitment strategy was adopted due to the absence of direct access to official migrant population records, given the confidentiality of the National Registry of Foreign Nationals under Chilean migration regulations ([Ley N° 21.325] Ministerio del Interior y Seguridad Pública, 2021). Although the sociodemographic characteristics of the sample are broadly consistent with available reports on Venezuelan migrants in Chile, the sample may not adequately represent migrants living in more vulnerable conditions or with different migration trajectories. In this regard, highly educated migrants may also experience specific forms of acculturative stress related to underemployment, professional deskilling, and difficulties in the recognition and homologation of academic and professional credentials in the host country.

Third, the temporal ordering of the variables should be interpreted with caution. Although psychological distress was assessed as recent symptom frequency during the previous week, whereas acculturative stress reflected broader migration-related experiences, the two-wave design does not allow definitive conclusions about causal directionality. In particular, because acculturative stress and psychological distress were both assessed at Time 2, the temporal precedence required to fully support mediation processes cannot be definitively established. In addition, the sensitivity analysis using a more temporally conservative specification did not support a robust prospective mediation process. Therefore, the findings should be interpreted as theoretically informed associations rather than as evidence of longitudinal mediation or causal mechanisms. Future research should use multi-wave longitudinal designs with repeated measures of the same constructs to test measurement invariance and examine directionality using cross-lagged panel models or similar approaches. Finally, data collection occurred during the COVID-19 pandemic, a context that may have intensified stress, uncertainty, and emotional distress among migrant populations.

Future research should consider mixed-methods approaches to deepen the understanding of adaptation and coping processes. Longer follow-up periods would allow researchers to examine more stable mental health trajectories. It would also be valuable to examine moderating variables such as coping strategies or perceived social support. Finally, including more diverse migrant groups would provide a broader view of the psychosocial dynamics involved in the migration experience. Despite these limitations, the present study contributes to the examination of associations between relatively stable dispositional traits, acculturative stress dimensions, and psychological distress over time among Venezuelan migrants in southern Chile. The findings may help inform the development of preventive and psychosocial interventions aimed at promoting migrant mental health and integration.

## Funding

This research was funded by the Agencia Nacional de Investigación y Desarrollo (ANID) through the program FONDECYT Iniciación Grant No. 11181020.

## CRediT authorship contribution statement

**María José Rivera-Baeza:** Writing – review & editing, Writing – original draft, Visualization, Methodology, Funding acquisition, Formal analysis, Conceptualization. **Camila Salazar-Fernández:** Writing – review & editing, Writing – original draft, Visualization, Methodology, Investigation, Formal analysis, Conceptualization. **Belén Salinas-Rehbein:** Writing – review & editing, Writing – original draft, Visualization, Methodology, Formal analysis, Conceptualization. **Paola Raipán-Gómez:** Writing – review & editing, Writing – original draft, Visualization.

## Declaration of competing interest

The authors declare that they have no known competing financial interests or personal relationships that could have appeared to influence the work reported in this paper.
